# Cytosolic phospholipase A_2_ contributes to innate immune defense against *Candida albicans* lung infection

**DOI:** 10.1186/s12865-016-0165-9

**Published:** 2016-08-08

**Authors:** Sabarirajan Jayaraja, Azzeddine Dakhama, Bogeon Yun, Moumita Ghosh, HeeJung Lee, Elizabeth F. Redente, Charis L. Uhlson, Robert C. Murphy, Christina C. Leslie

**Affiliations:** 1 Department of Pediatrics, National Jewish Health, 1400 Jackson St., Denver, Colorado 80206 USA; 2 Department of Pharmacology, University of Colorado Denver, Aurora, Colorado USA; 3 Department of Pathology, University of Colorado Denver, Aurora, Colorado USA

**Keywords:** Cytosolic phospholipase A_2_, Prostaglandins, Leukotrienes, Neutrophils, Macrophages, Inflammation, *Candida albicans*

## Abstract

**Background:**

The lung is exposed to airborne fungal spores, and fungi that colonize the oral cavity such as *Candida albicans*, but does not develop disease to opportunistic fungal pathogens unless the immune system is compromised. The Group IVA cytosolic phospholipase A_2_ (cPLA_2_α) is activated in response to *Candida albicans* infection resulting in the release of arachidonic acid for eicosanoid production. Although eicosanoids such as prostaglandins and leukotrienes modulate inflammation and immune responses, the role of cPLA_2_α and eicosanoids in regulating *C. albicans* lung infection is not understood.

**Methods:**

The responses of cPLA_2_α^+/+^ and cPLA_2_α^−/−^ Balb/c mice to intratracheal instillation of *C. albicans* were compared. After challenge, we evaluated weight loss, organ fungal burden, and the recruitment of cells and the levels of cytokines and eicosanoids in bronchoalveolar lavage fluid. The ability of macrophages and neutrophils from cPLA_2_α^+/+^ and cPLA_2_α^−/−^ mice to recognize and kill *C. albicans* was also compared.

**Results:**

After *C. albicans* instillation, cPLA_2_α^+/+^ mice recovered a modest weight loss by 48 h and completely cleared fungi from the lung by 12 h with no dissemination to the kidneys. In cPLA_2_α^−/−^ mice, weight loss continued for 72 h, *C. albicans* was not completely cleared from the lung and disseminated to the kidneys. cPLA_2_α^−/−^ mice exhibited greater signs of inflammation including higher neutrophil influx, and elevated levels of albumin and pro-inflammatory cytokines/chemokines (IL1α, IL1β, TNFα, IL6, CSF2, CXCL1, CCL20) in bronchoalveolar lavage fluid. The amounts of cysteinyl leukotrienes, thromboxane B_2_ and prostaglandin E_2_ were significantly lower in bronchoalveolar lavage fluid from *C. albicans*-infected cPLA_2_α^−/−^ mice compared to cPLA_2_α^+/+^ mice. Alveolar macrophages and neutrophils from uninfected cPLA_2_α^−/−^ mice exhibited less killing of *C. albicans* in vitro than cells from cPLA_2_α^+/+^ mice. In addition alveolar macrophages from cPLA_2_α^−/−^ mice isolated 6 h after instillation of GFP-*C. albicans* contained fewer internalized fungi than cPLA_2_α^+/+^ macrophages.

**Conclusions:**

The results demonstrate that cPLA_2_α contributes to immune surveillance and host defense in the lung to prevent infection by the commensal fungus *C. albicans* and to dampen inflammation.

**Electronic supplementary material:**

The online version of this article (doi:10.1186/s12865-016-0165-9) contains supplementary material, which is available to authorized users.

## Background

Group IVA cytosolic phospholipase A_2_ (cPLA_2_α) releases arachidonic acid to initiate eicosanoid production [1]. Eicosanoids are secreted and act locally through G-protein coupled receptors, which are expressed in a cell-type specific manner and initiate distinct signaling pathways to promote diverse biological responses [2–4]. Arachidonic acid is metabolized by 5-lipoxygenase (5-LO) to leukotrienes, and by constitutive cyclooxygenase (COX)-1 and inducible COX-2 to prostaglandins and thromboxane [5, 6]. Leukotrienes are pro-inflammatory mediators produced by macrophages, dendritic cells, mast cells, basophils and eosinophils that regulate cell trafficking, cytokine production, vascular permeability and phagocyte function [7]. The cysteinyl leukotrienes including leukotriene C_4_, leukotriene D_4_ and leukotriene E_4_ are bronchoconstrictors involved in asthma and allergic responses [2]. cPLA_2_α and COXs are widely expressed reflecting the ability of most cells and tissues to produce prostanoids, which have diverse functions [1, 6]. Prostaglandins regulate normal physiological processes such as female reproduction, hemostasis, kidney function and the maintenance of the gastrointestinal tract [1]. Although prostaglandins promote acute and chronic inflammation in response to tissue injury they also play a role in the resolution of inflammation and can be anti-inflammatory and immunosuppressive [8–10]. Therefore cPLA_2_α mediates the release of arachidonic acid for the production of numerous bioactive lipid mediators that have diverse effects [1]. This makes its role in regulating responses to infection difficult to predict and would be influenced by the specific tissue involved and nature of the microorganism.

Eicosanoids are produced rapidly in response to engagement of pattern recognition receptors by microbial pathogens and modulate immune cell function by affecting phagocytosis, microbial killing, chemotaxis and the transcriptional program [7, 10, 11]. We have used resident tissue macrophages from the peritoneal cavity and the lung to study the mechanisms of cPLA_2_α activation by the fungal pathogen *Candida albicans* [12–16]. Resident tissue macrophages are sentinel cells that are first responders to microbial invasion for initiating host defense to infection [17]. In resident peritoneal macrophages, activation of cPLA_2_α by *C. albicans* involves engagement of fungal cell wall polysaccharides β-glucan and mannans to C-type lectin receptors dectin-1 and dectin-2, respectively [13, 14]. These receptors act with MyD88-dependent pathways to activate cPLA_2_α, which involves calcium-induced translocation to membrane and phosphorylation by mitogen-activated protein kinases. In peritoneal macrophages, *C. albicans* stimulates an autocrine loop involving cPLA_2_α activation, production of prostaglandins and increases in cAMP that affects expression of genes involved in host defense and to dampen inflammation [15, 16]. In contrast, alveolar macrophages exhibit distinct properties since *C. albicans* poorly stimulates cPLA_2_α-mediated arachidonic acid release, however, priming with granulocyte macrophage colony-stimulating factor (GM-CSF) enhances arachidonic acid release by increasing expression of dectin-1 [12].

The lung has several mechanisms to clear environmental triggers that are continuously inhaled to prevent excess inflammation and tissue injury that may compromise gas exchange function [18]. Candida is the predominant fungal genus in the oral cavity, and dispersal of microoganisms from this site to the lung is a mechanism for shaping the lung microbiome [19, 20]. Despite potential exposure from the oral cavity, levels of *C. albicans* in the healthy lung are low indicating mechanisms for efficient clearance to prevent colonization [21, 22]. *C. albicans* is a commensal of mucosal surfaces that does not cause infection unless the immune system is compromised [23, 24]. Candida lung infection occurs in the critically ill, in patients with cancer and cystic fibrosis, during organ transplantation and in immune compromised individuals [21, 25, 26]. By comparing cPLA_2_α^+/+^ and cPLA_2_α^−/−^ mice, we found that cPLA_2_α contributes to innate immune defenses in the lung for protection against *C. albicans* infection.

## Methods

### Materials

Hank’s Balanced Salts Solution was from Invitrogen (Carlsbad, CA). ELISA kits were from eBioscience (San Diego, CA) (IL1α, IL1β, TNFα, IL6), from Immunology Consultants Laboratory Inc. (Portland, OR) (albumin), from R&D Systems (Minneapolis, MN) (CCL20) and from PeproTech (Rocky Hill, NJ) (CXCL1, CSF2, CSF3). Antibodies for flow cytometry analysis were from eBioscience (San Diego, CA) (anti-mouse CD45 eF450, CD11c PE, CD24 FITC, CD11b APC, MHC-II I-A/E PerCP-eF710, CD103 FITC) and from BD Biosciences (San Jose, CA) (anti-mouse Siglec F-PE and Ly6G (clone 1A8)-PE). QuickIII staining kit for cytospins was obtained from Astral Diagnostics, NJ. Butylated hydroxytoluene and indomethacin were from Fisher Scientific. Percoll, collagenase XI, Trypsin inhibitor, DNase I, RBC lysis solution were from Sigma-Aldrich (St. Louis, MO). Nylon cell strainers (70 μm) were from BD Biosciences (San Jose, CA). Qiasol lysis reagent, RNeasy Mini Kits and Mouse Cytokines & Chemokines RT^2^ Profiler PCR Array were from Qiagen (Valencia, CA). Paraformaldehyde was from Electron Microscopy Sciences (Hatfield, PA). XTT Cell Viability Kit was from Cell signaling.

### Mice

cPLA_2_α^−/−^ mice were generated as previously described [27], and backcrossed onto a Balb/c background for 10 generations. Balb/c control mice (cPLA_2_α^+/+^) were obtained from Charles River (San Diego, CA). Mice were housed under specific pathogen free conditions and used between 8–14 weeks of age. Male mice were used for all experiments with exception as noted in the figure legend. The work with mice was approved by the Institutional Animal Care and Use Committee (IACUC) at National Jewish Health and conducted in accordance with their guidelines.

### *C. albicans* challenge

*C. albicans* (ATCC SC5314) was grown in YPD medium overnight (30 °C), washed, suspended in endotoxin-free PBS then counted. Counts correlated directly with colony forming units (CFU). *C. albicans* was administered by intratracheal instillation to cPLA_2_α^+/+^ and cPLA_2_α^−/−^ Balb/c mice under isoflurane anesthesia. The trachea was intubated with a gavage needle to instill (50 μl) *C. albicans* (10^6^–10^7^ CFU) or endotoxin-free PBS. Mice were euthanized by CO_2_ asphyxiation or cervical dislocation with similar results. *C. albicans* expressing green fluorescent protein (GFP) was kindly provided by Dr. Robert Wheeler, The University of Maine. It was generated from the wild type SC5314 strain and exhibits similar virulence as the wild type strain in mice [28].

### Bronchoalveolar lavage

Lungs were lavaged 5 times as described [12]. For analysis of eicosanoids in bronchoalveolar lavage fluid (BALF), the lavage solution also contained 5 μM indomethacin and 50 μM butylated hydroxytoluene. Cells in lavage were differentiated on cytospins. Albumin, cytokines and chemokines were measured in BALF by ELISA.

### Fungal burden

Blood was drained by cutting the inferior vena cava, and then lungs and kidneys were removed asceptically, weighed and homogenized (Omni Tissue Homogenizer, Omni International) in sterile phenol red-free HBSS. Homogenates were serially diluted, plated on Sabouraud dextrose agar plates containing penicillin and streptomycin, and then *C. albicans* CFU determined after 48 h incubation at 37 °C.

### Histology

Lungs were fixed by inflation (1 ml), immersed in formalin (10 %) then dehydrated and embedded in paraffin. Sections (5 μm) were stained with H & E.

### Real-time PCR

Lungs from cPLA_2_α^+/+^ and cPLA_2_α^−/−^ mice were homogenized with an Omni Tissue Homogenizer in Qiasol lysis reagent and RNA isolated using on-column DNase treatment. RNA concentration and purity were determined by UV spectrophotometry, and RNA integrity verified using an Agilent Bioanalyzer 2100. cDNA was synthesized from RNA (200 ng) using RT^2^ First Strand Kit (Qiagen). Real-time PCR was performed using RT^2^ qPCR Mastermix and a Mouse Cytokines & Chemokines RT^2^ Profiler PCR Array according to the manufacturer's protocol using the StepOnePlus Real-Time PCR System (Applied Biosystems). RT^2^ PCR arrays in a 96-well format were used containing pre-validated primers tested for efficiency (Qiagen). The RT^2^ Profiler PCR Array System included a reverse transcription control preloaded into the primer buffer of the RT^2^ First Strand cDNA synthesis kit that measured the relative efficiency of the reverse transcription for all the samples. A genomic DNA control and a positive PCR control were also included in the system. The RT^2^ Profiler PCR Array data were normalized to the housekeeping gene *Gusb* and the relative gene expression level (2^(−ΔC_t_) was calculated using the formula ΔC_t_ = C_t_ (gene of interest)- C_t_ (housekeeping gene). The data were analyzed on the PCR array data analysis SA Biosciences web portal (http://pcrdataanalysis.sabiosciences.com/pcr/arrayanalysis.php).

Real-time PCR was also performed with cDNA synthesized with random hexamer primers (Fermentas Maxima First Strand cDNA Synthesis Kit, Thermo Scientific) using TaqMan fast universal PCR master mix. TaqMan assay probes used were: *Clec7a* (dectin-1) (Mm01183349_m1), *Clec4n* (dectin-2) (Mm00490934_m1) and *Gusb* (Mm01197698_g1). The housekeeping gene *Gusb* was used for normalization. Threshold cycle values (*C*_*T*_) were determined and used for ∆∆C_T_ analysis of gene expression [29].

### Lung digestion and flow cytometry analysis

After performing bronchoalveolar lavage, blood was drained from the lungs by cutting the inferior vena cava. Lungs were removed, cut into small pieces followed by digestion with 5 ml collagenase solution (0.5 mg/ml collagenase XI, 0.2 mg/ml trypsin inhibitor, 5 % FBS in minimum essential medium) for 1 h at 37 °C with occasional mixing. The digested lungs were sheared with an 18-gauge needle, treated with 50 μl of DNase I solution (5 mg/ml) and then incubated for 10 min at 37 °C. Lung digests were filtered through 70-μm nylon cell strainers and the single cell suspension treated with RBC lysis solution. Cells were counted using a Countess cell counter (Invitrogen, Carlsbad, CA) excluding dead cells with trypan blue. Cells were resuspended in flow cytometry (FC) buffer (2 % FBS, 0.1 % BSA, 0.05 % sodium azide in PBS) at 2 × 10^6^ cells/ml. All the steps were done at 4 °C. Cells were dispensed (0.5 x 10^6^ cells in 250 μl) in V-shaped 96 well plates. After centrifugation at 1500 rpm for 5 min, the supernatant was removed and 50 μl of FcBlock (anti-CD16/CD32, clone 2.4G2, 40 μg/ml in FC buffer, eBiosciences) was added followed by incubation on ice for 15 min. Cells were then treated with 50 μl of antibody cocktails, incubated on ice for 30 min followed by addition of 150 μl FC buffer then washed in FC buffer. Cells were fixed with 4 % paraformaldehyde in PBS (100 μl/well), pH 7.4, then transferred to FC tubes in 300 μl FC buffer and stored in the dark at 4 °C until analysis. Data were acquired on a Dako Cyan ADP flow cytometer. Compensation and data analyses were performed using FlowJo software (TreeStar, Ashland, OR). After the exclusion of doublets and debris, immune cells were identified by CD45 positive staining. A sequential gating strategy was used to identify cell populations: alveolar macrophages (CD45^+^ CD24^−^ CD11b^−^ SiglecF^+^); tissue macrophages (CD45^+^ CD24^−^ CD11b^+^); neutrophils (CD45^+^ CD11b^+^ Ly6G^+^) and CD11b^+^ dendritic cells (CD11b^+^ DCs) (CD45^+^ MHCII^+^ CD11c^+^ CD11b^+^) (Additional file 1) [30].

### Eicosanoid measurements

BALF stored at −80 °C was thawed and mixed with an equal volume of cold methanol. Just before analysis, the samples were diluted in water to a final methanol concentration of less than 15 % and then extracted using a solid phase extraction cartridge (Strata Polymeric Reverse Phase 60 mg/ml; Phenomenex, Torrance, CA). The eluate (1 ml of methanol) was dried and reconstituted in 75 μl of high-performance liquid chromatography (HPLC) solvent A (8.3 mM acetic acid buffered to pH 5.7 with NH_4_OH) and 25 μl of solvent B (acetonitrile/methanol, 65/35, v/v). An aliquot of each sample (30 μl) was injected into an HPLC and metabolites separated on a C18 column (Kinetex EVO C18 100A 50 x 3.0 mm, 5 μm; Phenomenex, Torrance, CA) eluted at a flow rate of 0.25 ml/min with a linear gradient from 25 % to 75 % solvent B in 13 min then increased to 98 % in 2 min and held for 11 min. The HPLC system was directly interfaced into the electrospray ionization source of a triple quadrapole mass spectrometer (Sciex API 5500; PE-Sciex, Thornhill, ON, Canada). Mass spectrometric analyses were performed in the negative ion mode using multiple reaction monitoring of the specific transitions: [d4]PGE_2_*m/z* 355 → 275, [d4]PGD_2_*m/z* 355➔237, [d4]TXB_2_*m/z* 373 → 173, [d4]6-keto-PGF_1_α *m/z* 373➔167, [d5]LTC_4_*m/z* 629 → 271, [d5]LTD_4_*m/z* 500➔177, [d5]LTE_4_*m/z* 443➔338, PGE_2_*m/z* 351 → 271, PGD_2_*m/z* 351➔233, TXB_2_*m/z* 369 → 169, 6-Keto-PGF_1_α *m/z* 369➔ 163, LTC_4_*m/z* 624 → 272, LTD_4_*m/z* 495➔177, LTE_4_*m/z* 438➔333. Quantitation was performed using a standard isotope dilution curve as described [31].

### *C. albicans* recognition and killing assays

Alveolar macrophages were isolated from untreated cPLA_2_α^+/+^ and cPLA_2_α^−/−^ mice by lavage and cultured as previously described [12]. Live opsonized and unopsonized GFP-*C. albicans* (moi 2) was used for all assays. GFP-*C. albicans* was opsonized by incubating in DMEM containing 10 % mouse serum for 30 min at 37 °C before incubation with the macrophages. For evaluating binding and internalization (recognition assay), alveolar macrophages (1 × 10^5^) were seeded onto the glass insert of MatTek 35 mm dishes and incubated for 2 h [12]. Cells were washed then incubated with GFP*-C. albicans* in phenol red-free DMEM containing penicillin, streptomycin and 0.1 % endotoxin-free BSA (stimulation media) for 30 min at 37 °C and 5 % CO_2_. Macrophages were washed, fixed with 4 % paraformaldehyde for 15 min and then stained with DAPI. Images were captured on a Marianas 200 spinning disk confocal microscope using Intelligent Imaging Innovation Inc. (3I) software (Slidebook 6.0) to determine the number of macrophages containing GFP-*C albicans*. For killing assays, alveolar macrophages (in 48 well plates) were incubated for 2 h in stimulation media with GFP-*C. albicans*. Wells containing an equivalent number of GFP-*C. albicans* (without macrophages) were included as a positive control for determining 100 % viability. Macrophages were lysed with 1 % Triton X-100 and GFP-*C. albicans* viability was measured using the XTT Cell Viability Kit as described [32].

Bone marrow neutrophils were isolated from untreated cPLA_2_α^+/+^ and cPLA_2_α^−/−^ mice as described previously and purity (>95 %) determined on cytospins [33]. Neutrophils (1 × 10^5^) were plated on polylysine-coated MatTek 35 mm dishes, incubated for 1 h and then incubated with GFP*-C. albicans* for 30 min. After fixation the cells were incubated for 1 h in PBS containing 10 % FBS and then incubated overnight with anti-Ly6G antibody followed by treatment with anti-rabbit AF594 secondary antibody and with DAPI. For killing assays, GFP-*C. albicans* was added to neutrophils (5 × 10^4^) in the 96 well plates, centrifuged for 5 min at 300 g to synchronize the infection, and then incubated for 2 h at 37 °C and 5 % CO_2_. GFP-*C. albicans* viability was determined as described above for macrophages.

### Statistics

The data are presented as mean ± SEM and analyzed using the 2-tailed unpaired *t*-test or the Mann Whitney method to determine statistical significance (defined as *p* < 0.05).

## Results

### *C. albicans* infection causes greater weight loss in cPLA_2_α^−/−^ than cPLA_2_α^+/+^ mice

**Fig. 1:**
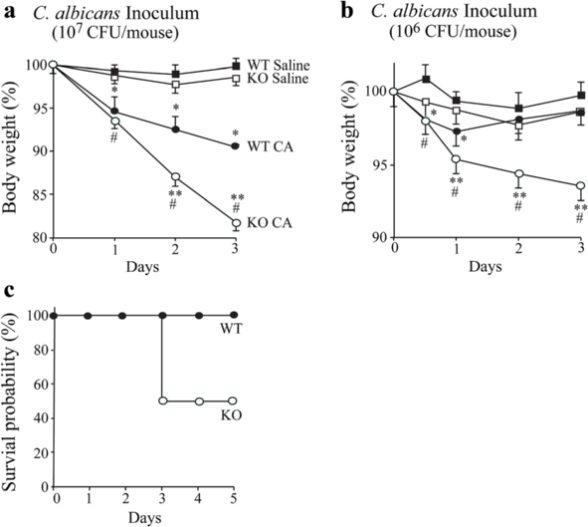
Weight loss is greater in cPLA_2_α^−/−^ than cPLA_2_α^+/+^ mice during *C. albicans* lung infection. Body weight of cPLA_2_α^−/−^ (KO, open symbols) and cPLA_2_α^+/+^ (WT, closed symbols) mice was monitored after intratracheal instillation of saline (squares) or *C. albicans* (CA, circles) using an inoculum of (**a**) 10^7^ or (**b**) 10^6^ CA. Body weight is expressed as the % of the weight determined just prior to instillation of *C. albicans* or saline (*n* = 8–13 mice/group, from 4–6 independent experiments). *P < 0.05 compared to cPLA_2_α^+/+^ with saline; #*P* < 0.05 compared to cPLA_2_α^−/−^ with saline; **P < 0.05 compared to cPLA_2_α^+/+^ with CA. **c** Survival of female cPLA_2_α^−/−^ (KO, open symbols) and cPLA_2_α^+/+^ (WT, closed symbols) mice was monitored after intratracheal administration of 5 x 10^6^ CFU *C. albicans* (6 mice/group)

The role of cPLA_2_α in regulating host defense against *C. albicans* lung infection was investigated by comparing responses of cPLA_2_α^+/+^ and cPLA_2_α^−/−^ Balb/c mice. The LD_50_ from intratracheal challenge with *C. albicans* in immune competent mice is approximately 10^8^ CFU [34]. We first determined if concentrations below the LD_50_ (10^6^ and 10^7^ CFU) induced weight changes (Fig. 1). There was significant weight loss from both cPLA_2_α^+/+^ and cPLA_2_α^−/−^ mice compared to saline controls 24 h after instillation of 10^7^ Candida that continued for 72 h (Fig. 1a). Weight loss was significantly greater from cPLA_2_α^−/−^ than cPLA_2_α^+/+^ mice at 48 and 72 h. Challenging mice with 10^6^*C. albicans* resulted in a small but significant weight loss in cPLA_2_α^+/+^ mice at 12 and 24 h compared to saline controls followed by recovery of normal weight by 48–72 h (Fig. 1b). In cPLA_2_α^−/−^ mice challenged with 10^6^*C. albicans,* weight loss continued from 24–72 h and was significantly greater than in cPLA_2_α^+/+^ mice. cPLA_2_α^+/+^ and cPLA_2_α^−/−^mice were challenged with an intermediate amount of *C. albicans* (5 × 10^6^ CFU) and survival and body weight monitored for 28 days. All cPLA_2_α^+/+^ mice survived but 50 % of cPLA_2_α^−/−^ mice died by 72 h with no further mortality for 28 days (Fig. 1c). cPLA_2_α^−/−^ mice lost significantly more weight (16.5 % ± 1.1), which was greatest at day 4, than cPLA_2_α^+/+^ mice (6.5 % ± 0.4). The surviving cPLA_2_α^−/−^ mice and all cPLA_2_α^+/+^ mice started gaining weight after day 4 that returned to normal by ~18 days. Since the results suggest that cPLA_2_α regulates early host defense to *C. albicans* lung infection, we focused on comparing early responses of cPLA_2_α^+/+^ and cPLA_2_α^−/−^ mice up to 72 h after *C. albicans* challenge.

### *C. albicans* is not cleared completely from the lungs of cPLA_2_α^−/−^ mice and disseminates to the kidney

**Fig. 2:**
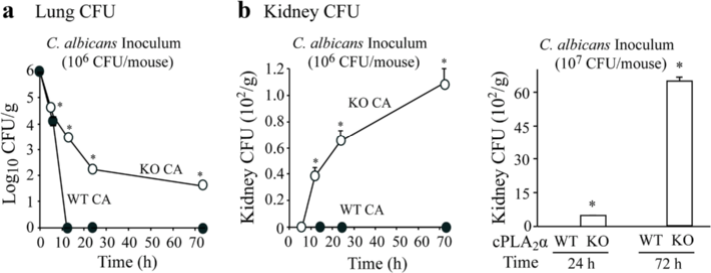
*C. albicans* is not cleared completely from lungs of cPLA_2_α^−/−^ mice and disseminates to the kidney. cPLA_2_α^−/−^ (open circles) and cPLA_2_α^+/+^ (closed circles) mice were challenged with either 10^6^ or 10^7^ *C. albicans* (CA), and CFU were determined at the indicated times in homogenized (**a**) lung and (**b**) kidney (*n* = 3–14 mice/group, from 3–6 independent experiments). *P < 0.05 compared to cPLA_2_α^+/+^

The ability of cPLA_2_α^+/+^ and cPLA_2_α^−/−^ mice to clear *C. albicans* was compared by measuring fungal CFU in lung homogenates. It has previously been reported that immune competent mice are resistant to infection and rapidly eliminate *C. albicans* from the lung [34, 35]. Analysis of lungs 5 min after intratracheal challenge with 10^6^*C. albican*s confirmed that greater than 90 % of the inoculum delivered to the lung was recovered in homogenates. By 6 h after instillation most (≥98 %) of the *C. albicans* was cleared from the lungs of cPLA_2_α^+/+^ and cPLA_2_α^−/−^ mice although significantly more remained in cPLA_2_α^−/−^ mice than cPLA_2_α^+/+^ mice (Fig. 2a). cPLA_2_α^+/+^ mice completely cleared *C. albicans* from the lung with no viable fungi recovered from 12–72 h after instillation, whereas a significant fungal burden persisted in cPLA_2_α^−/−^ mice during this time period. Using a higher inoculum (10^7^ CFU), a low level of *C. albicans* was recovered in lungs of cPLA_2_α^+/+^ mice (150 ± 14 CFU/g) at 72 h, and 30-fold higher levels in cPLA_2_α^−/−^ mice (4567 ± 450 CFU/g). We also determined if *C. albicans* breached the lung and disseminated to the kidney, which is the primary target organ in mice and humans in disseminated candidiasis [36, 37]. *C. albicans* was recovered from the kidneys of cPLA_2_α^−/−^ mice challenged with 10^6^ CFU at 12 h that further increased from 24–72 h (Fig. 2b). Using an inoculum of 10^7^ CFU, the kidneys of cPLA_2_α^−/−^ mice contained considerably more *C. albicans* than the relatively low level in mice challenged with 10^6^ CFU. In contrast *C. albicans* was not detected in kidneys of cPLA_2_α^+/+^ mice challenged with 10^6^ or 10^7^*C. albicans* (Fig. 2b). The results demonstrate a critical protective role for cPLA_2_α in the early stages of Candida clearance and dissemination in vivo. Based on these results we investigated differences in the early host defense responses in cPLA_2_α^+/+^ and cPLA_2_α^+/+^ mice using the lower inoculum of 10^6^*C. albicans*.

### cPLA_2_α^−/−^ mice have higher numbers of neutrophils in BALF and lung tissue than cPLA_2_α^+/+^ mice during *C. albicans* infection

**Fig. 3:**
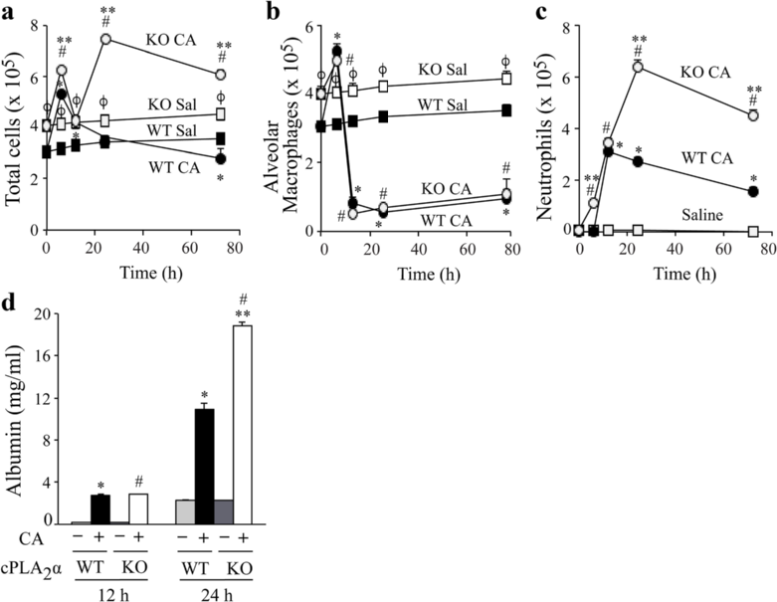
Neutrophils and albumin are higher in BALF of cPLA_2_α^−/−^ than cPLA_2_α^+/+^ mice during *C. albicans* infection. The number of (**a**) total cells, (**b**) alveolar macrophages and (**c**) neutrophils were determined in BALF (5 lavages) from cPLA_2_α^−/−^ (KO, open symbols) and cPLA_2_α^+/+^ (WT, closed symbols) mice instilled with saline (Sal, squares) or 10^6^ *C. albicans* (CA, circles). **d** cPLA_2_α^+/+^ (WT) and cPLA_2_α^−/−^ (KO) mice were lavaged 12 and 24 h after instillation of 10^6^ *C. albicans* or saline. Albumin levels were determined in BALF by ELISA. (*n* = 8-13 mice/group, from 4–6 independent experiments). *P < 0.05 compared to cPLA_2_α^+/+^ saline control; ^ϕ^ *P* < 0.05 compared to cPLA_2_α^+/+^ saline control, ^#^ *P* < 0.05 compared to cPLA_2_α^−/−^ saline control; **P < 0.05 compared to cPLA_2_α^+/+^ with CA

To evaluate the extent of inflammation in cPLA_2_α^+/+^ and cPLA_2_α^−/−^ mice, the recruitment of cells into BALF was compared from 6–72 h after intratracheal instillation of 10^6^*C. albicans* or saline (Fig. 3). The number of total cells recovered in BALF of untreated (0-time) and saline control mice was significantly higher (~20 %) in cPLA_2_α^−/−^ than cPLA_2_α^+/+^ mice that was due to higher numbers of alveolar macrophages (Fig. 3a and b). Over 95 % of the cells in BALF of cPLA_2_α^+/+^ and cPLA_2_α^−/−^ control mice were alveolar macrophages. The number of total cells in BALF increased 6 h after *C. albicans* challenge to a slightly greater level in cPLA_2_α^−/−^ mice, then decreased by 12 h to similar levels in cPLA_2_α^+/+^ and cPLA_2_α^−/−^ mice (Fig. 3a). Between 12 and 24 h after *C. albicans* instillation the number of total cells in cPLA_2_α^+/+^ mice slightly declined but increased in cPLA_2_α^−/−^ mice due to greater neutrophil influx (Fig. 3a and c). The number of alveolar macrophages in BALF increased at 6 h after *C. albicans* challenge to similar levels in cPLA_2_α^+/+^ and cPLA_2_α^−/−^ mice followed by a sharp decline in both strains at 12 h that remained low for 72 h (Fig. 3b). The decrease in the number of alveolar macrophages is reminiscent of the macrophage disappearance reaction observed in response to inflammation in the peritoneal cavity that is attributed to macrophage activation resulting in increased adherence or trafficking [17, 38]. Neutrophils significantly increased in BALF from cPLA_2_α^−/−^ but not cPLA_2_α^+/+^ mice 6 h after *C. albicans* instillation but then increased to similar levels in both strains at 12 h (Fig. 3c). Neutrophil numbers in cPLA_2_α^+/+^ mice peaked at 12 h but continued to increase in cPLA_2_α^−/−^ mice up to 24 h reaching levels 2.3-fold higher than in cPLA_2_α^+/+^ mice. Neutrophil numbers in cPLA_2_α^−/−^ mice remained >2-fold higher than the levels in cPLA_2_α^+/+^ mice up to 72 h after infection (Fig. 3c). It has been demonstrated that neutrophil influx into the lung during bacterial pneumonia contributes to alveolar barrier disruption promoting leakage of plasma protein into the alveolar space [39]. To determine if the increased neutrophil influx in *C. albicans*-infected cPLA_2_α^−/−^ mice was accompanied by an increase in protein leak into the lung, the amount of albumin in BALF was determined by ELISA (Fig. 3d). Albumin levels increased at 12 h after *C. albicans* instillation to a similar extent in cPLA_2_α^−/−^ than cPLA_2_α^+/+^ mice. Albumin levels increased by 24 h and were 1.8-fold higher in cPLA_2_α^−/−^ mice compared to cPLA_2_α^+/+^ mice. The results indicate a greater compromise of alveolar barrier function in cPLA_2_α^−/−^ mice that correlated with a higher level of neutrophil influx in response to *C. albicans* infection.

**Fig. 4:**
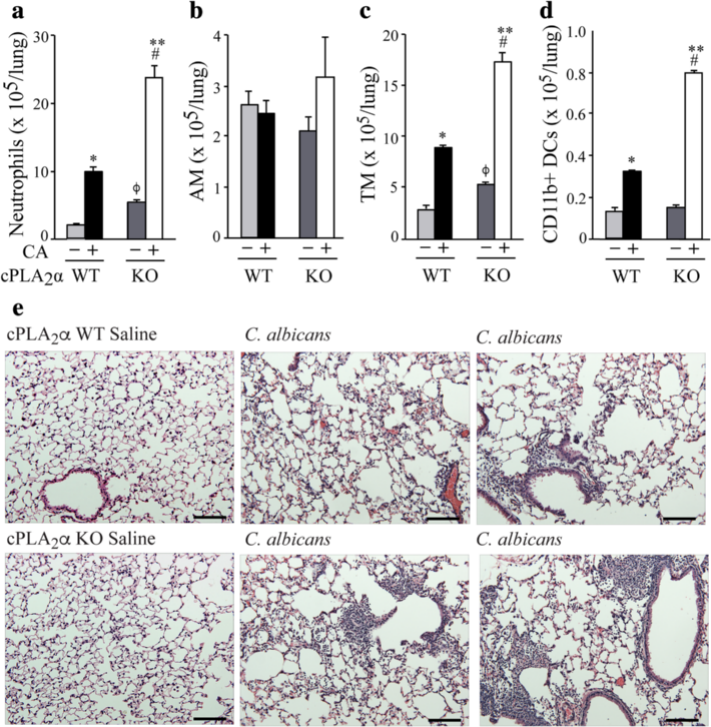
Effect of *C. albicans* infection on cell composition and histopathology of lung tissue from cPLA_2_α^+/+^ and cPLA_2_α^−/−^ mice. cPLA_2_α^−/−^ (KO) and cPLA_2_α^+/+^ (WT) mice were challenged with saline or 10^6^ CFU *C. albicans* (CA) for 24 h and then lavaged. Lung tissue was processed for identification of (**a**) Neutrophils, (**b**) alveolar macrophages (AM), (**c**) tissue macrophages (TM) and (**d**) CD11b^+^ dendritic cells (DC) by flow cytometry (n = 3 mice/group). *P < 0.05 compared to cPLA_2_α^+/+^ saline control; ^ϕ^ *P* < 0.05 compared to cPLA_2_α^+/+^ saline control, ^#^ *P* < 0.05 compared to cPLA_2_α^−/−^ saline control; **P < 0.05 compared to cPLA_2_α^+/+^ with CA. **e** Representative images of H & E stained lung sections from saline controls and from *C. albicans*-infected cPLA_2_α WT or cPLA_2_α KO mice are shown*.* Scale bar = 100 μm

Cell influx into lung tissue of cPLA_2_α^+/+^ and cPLA_2_α^−/−^ mice 24 h after *C. albicans* infection was also evaluated by flow cytometry (Fig. 4). *C. albicans* infection stimulated a significant increase in neutrophils (CD45^+^CD11b^+^Ly6G^+^) in lung tissue of both cPLA_2_α^+/+^ and cPLA_2_α^−/−^ mice but numbers were 2.5-fold higher in cPLA_2_α^−/−^ mice (Fig. 4a). There were similar numbers of alveolar macrophages (CD45^+^CD24^−^CD11b^−^SiglecF^+^) in lungs of cPLA_2_α^+/+^ and cPLA_2_α^−/−^ mice, and numbers were not affected by *C. albicans* infection (Fig. 4b). This suggests that the higher number of alveolar macrophages in BALF of uninfected cPLA_2_α^−/−^ mice (see Fig. 3a) may be due to differences in adherence properties that influence their recovery by lavage. *C. albicans* infection stimulated an increase in tissue macrophages (CD45^+^CD24^−^CD11b^+^), which were significantly higher in cPLA_2_α^−/−^ compared to cPLA_2_α^+/+^ mice (Fig. 4c). The tissue macrophage population, which includes both interstitial macrophages and monocytes, may increase due to recruitment of monocytes from the blood in response to *C. albicans* infection. CD11b^+^ dendritic cells (CD45^+^MHCII^+^CD11c^+^CD11b^+^) increased in response to *C. albicans* infection to a greater extent in cPLA_2_α^−/−^ compared to cPLA_2_α^+/+^ mice (Fig. 4d). Representative histograms of the flow cytometry analysis are shown in Additional file 1. Histological examination of lung sections 24 h after *C. albicans* challenge showed little evidence of inflammation other than an occasional small patch of focal inflammation in cPLA_2_α^+/+^ mice. The patches of inflammation were markedly larger and more extensive in cPLA_2_α^−/−^ than in cPLA_2_α^+/+^ mice (Fig. 4e).

### cPLA_2_α influences gene expression and cytokine production in lungs of *C. albicans* infected mice

We previously reported that activation of cPLA_2_α in *C. albicans*-infected macrophages influences gene expression through an autocrine loop involving the production of prostaglandins and increases in cAMP [15, 16]. We first screened differences in gene expression in total lung tissue of cPLA_2_α^+/+^ and cPLA_2_α^−/−^ mice at 12 and 24 h after instillation of *C. albicans* or saline by using a cytokine/chemokine PCR array (Additional file 2). *C. albicans* infection stimulated an increase in expression of several pro-inflammatory cytokines (*Il1α*, *Il1β*, *Tnfα*, *Il6*), and the immune mediators *Csf2* and *Ccl20,* in lungs of cPLA_2_α^+/+^ and cPLA_2_α^−/−^ mice*.* The level of these cytokines was significantly higher in cPLA_2_α^−/−^ compared to cPLA_2_α^+/+^mice particularly 12 h after *C. albicans* challenge. The chemokines *Ccl2*, *Ccl7* and *Cxcl1* were also expressed at higher levels in cPLA_2_α^−/−^ compared to cPLA_2_α^+/+^mice 12 h after infection, but at 24 h they decreased to a greater extent in cPLA_2_α^−/−^ than cPLA_2_α^+/+^mice. *Cxcl10* and *Ccl12* increased during *C. albicans* infection to the same extent in cPLA_2_α^+/+^ and cPLA_2_α^−/−^ mice at 12 h but were significantly lower in cPLA_2_α^−/−^ than cPLA_2_α^+/+^mice at 24 h. The results evaluating gene expression in the total lung suggested that cPLA_2_α activation suppresses the expression of several pro-inflammatory cytokines but also influences the duration of gene expression particularly for certain chemokines (*Ccl2, Ccl7, Ccl12, Cxcl1, Cxcl10*).

**Fig. 5:**
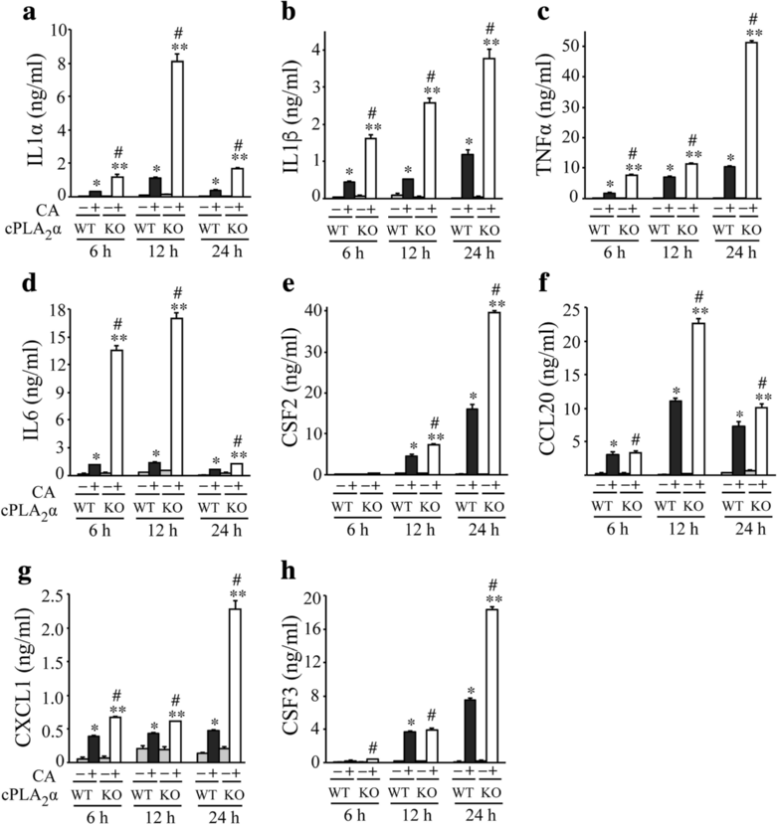
Pro-inflammatory cytokines and chemokines are higher in BALF from cPLA_2_α^−/−^ than cPLA_2_α^+/+^ mice during *C. albicans* infection. cPLA_2_α^−/−^ (KO) and cPLA_2_α^+/+^ (WT) mice were challenged with saline or 10^6^ *C. albicans* (CA) for 6, 12 and 24 h. The levels of (**a**) IL1α, (**b**) IL1β, (**c**) TNFα, (**d**) IL6, (**e**) CSF2, (**f**) CCL20, (**g**) CXCL1 and (**h**) CSF3 were determined by ELISA (*n* = 6–10 mice/group in 3–5 experiments). *P < 0.05 compared to cPLA_2_α^+/+^ saline control; ^#^ *P* < 0.05 compared to cPLA_2_α^−/−^ saline control; **P < 0.05 compared to cPLA_2_α^+/+^ with CA

Since *C. albicans* first encounters cells lining the airways and in the alveoli, cytokines and chemokines were measured in BALF from cPLA_2_α^+/+^ and cPLA_2_α^−/−^ mice collected 6–24 h after *C. albicans* infection (Fig. 5). The pro-inflammatory cytokines IL1α (Fig. 5a), IL1β (Fig. 5b), TNFα (Fig. 5c) and IL6 (Fig. 5d) were significantly higher in BALF of cPLA_2_α^−/−^ compared to cPLA_2_α^+/+^ mice at all time points but the time of peak production differed. IL1α production was transient and peaked at 12 h in cPLA_2_α^−/−^ mice reaching levels that were 8-fold higher than in cPLA_2_α^+/+^ mice (Fig. 5a). TNFα continued to increase in cPLA_2_α^−/−^ mice for 24 h (Fig. 5c). IL6 was 10-fold higher in cPLA_2_α^−/−^ compared to cPLA_2_α^+/+^ mice at 6 and 12 h after infection then decreased by 24 h (Fig. 5d). There was early production of IL1β in cPLA_2_α^−/−^ mice that continued to increase up to 24 h after *C. albicans* infection (Fig. 5b). CSF2 (Fig. 5e) and CCL20 (Fig. 5f) were significantly higher in cPLA_2_α^−/−^ than cPLA_2_α^+/+^mice at 12 and 24 h after infection. The neutrophilic chemokine CXCL1 was higher in BALF of cPLA_2_α^−/−^ than cPLA_2_α^+/+^ mice particularly 24 h after *C. albicans* infection (Fig. 5g). Although levels of *Csf3* mRNA were similar in lungs of *C. albicans* infected cPLA_2_α^−/−^ and cPLA_2_α^+/+^mice, analysis of BALF showed that CSF3 was higher in cPLA_2_α^−/−^ than cPLA_2_α^+/+^ mice at 24 h (Fig. 5h). The results demonstrate that cPLA_2_α^−/−^ mice have higher levels of pro-inflammatory cytokines and chemokines consistent with increased neutrophil recruitment.

### Levels of eicosanoids in BALF from cPLA_2_α^+/+^ and cPLA_2_α^−/−^ mice during *C. albicans* infection

**Fig. 6:**
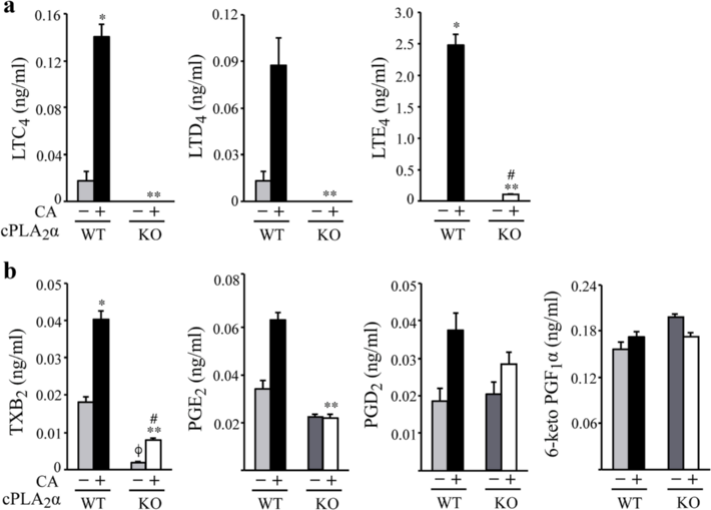
Eicosanoids are lower in BALF from cPLA_2_α^−/−^ than cPLA_2_α^+/+^ mice during *C. albicans* infection. Levels of (**a**) cysteinyl leukotrienes, leukotriene C_4_ (LTC_4_), leukotriene D_4_ (LTD_4_) and leukotriene E_4_ (LTE_4_) and (**b**) cyclooxygenase metabolites thromboxane B_2_ (TXB_2_), prostaglandin E_2_ (PGE_2_), prostaglandin D_2_ (PGD_2_) and 6-keto prostaglandin F_1_α (6-keto PGF_1_α) were determined by mass spectrometry in BALF from cPLA_2_α^−/−^ (KO) and cPLA_2_α^+/+^ (WT) mice instilled with saline or 10^6^ *C. albicans* (CA) for 24 h. Results are the mean ± SEM (*n* = 6 mice/group). Statistical differences were determined by the Mann Whitney protocol. *P < 0.05 compared to WT saline control; ^ϕ^ *P* < 0.05 compared to WT saline control, ^#^ *P* < 0.05 compared to KO saline control; **P < 0.05 compared to WT with CA

cPLA_2_α releases arachidonic acid for production of eicosanoids, which play diverse roles in regulating inflammation and innate immunity. Eicosanoids were analyzed by mass spectrometry in BALF collected 24 h after *C. albicans* infection from cPLA_2_α^+/+^ and cPLA_2_α^−/−^ mice (Fig. 6). Since cyclooxygenase metabolites and oxidation products can be generated during tissue processing from available free arachidonic acid, the cyclooxygenase inhibitor indomethacin and antioxidant butylated hydroxytoluene were added to the lavage solution before administration. By including D8-arachidonic acid in the lavage solution along with indomethacin, preliminary experiments showed that cyclooxygenase products were not generated during the lavage procedure since D8 metabolites were not found. In addition we found that it was necessary to include butylated hydroxytoluene during lavage to prevent the formation of isoprostanes. As shown in Fig. 6, *C. albicans* stimulated an increase in cysteinyl leukotriene production in cPLA_2_α^+/+^ mice with the stable metabolite leukotriene E_4_ being the most abundant followed by leukotriene C_4_ and leukotriene D_4_ (Fig. 6a). Cysteinyl leukotrienes were at very low or undetectable levels in saline controls and in BALF from *C. albicans* infected cPLA_2_α^−/−^ mice indicating that cPLA_2_α initiates their production. There was no significant production of leukotriene B_4_ in saline controls or in response to *C. albicans* infection in either cPLA_2_α^+/+^ or cPLA_2_α^−/−^ mice. The cyclooxygenase metabolites thromboxane B_2_ (the stable metabolite of thromboxane A_2_), prostaglandin E_2_ and prostaglandin D_2_ were detected at the highest levels in BALF of *C. albicans* infected cPLA_2_α^+/+^ mice (Fig. 6b). Thromboxane B_2_ and Prostaglandin E_2_ were significantly lower in *C. albicans*-infected cPLA_2_α^−/−^ than cPLA_2_α^+/+^ mice. Although prostaglandin D_2_ levels were lower in *C. albicans*-infected cPLA_2_α^−/−^ mice than cPLA_2_α^+/+^ mice this did not reach statistical significance. The stable metabolite of prostaglandin I_2_ (6-keto-prostaglandin F_1_α) was detected in BALF at relatively high endogenous levels but was not increased by *C. albicans* and not significantly different in cPLA_2_α^+/+^ and cPLA_2_α^−/−^ mice (Fig. 6b). Therefore the higher levels of cysteinyl leukotrienes, thromboxane A_2_ or prostaglandin E_2_ in cPLA_2_α^+/+^ than cPLA_2_α^−/−^ mice may be important for protecting the lung against *C. albicans* infection.

### Functional differences in alveolar macrophages and neutrophils from cPLA_2_α^+/+^ and cPLA_2_α^−/−^ mice

**Fig. 7:**
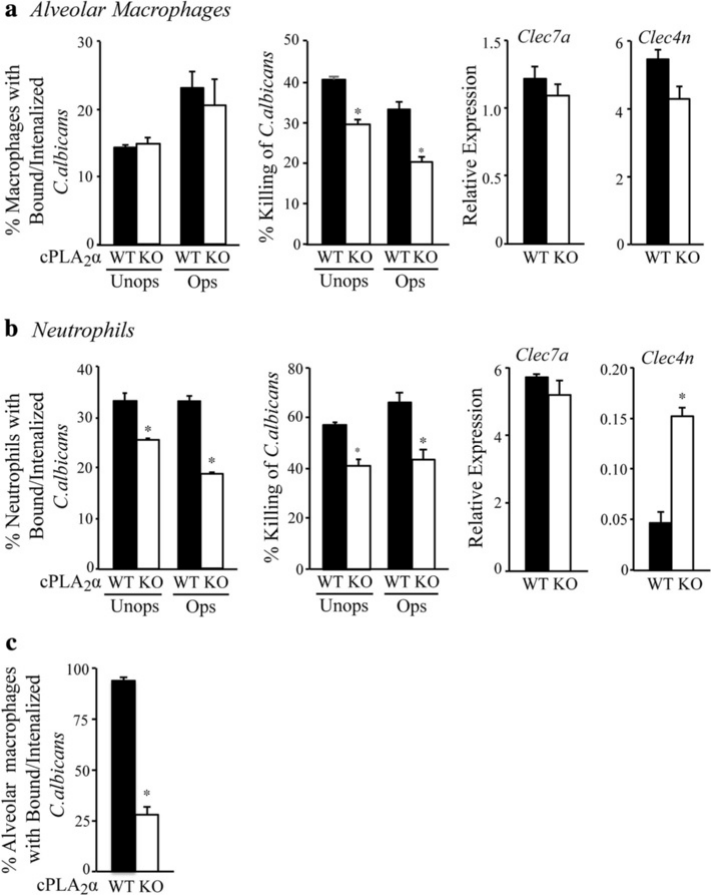
Recognition and killing of *C. albicans* by alveolar macrophages and neutrophils from cPLA_2_α^−/−^ and cPLA_2_α^+/+^ mice. The ability of (**a**) alveolar macrophages and (**b**) neutrophils from untreated cPLA_2_α^+/+^ (WT) and cPLA_2_α^−/−^ (KO) mice to recognize and kill GFP-*C. albicans* in vitro was compared. Levels of *clec7a* (dectin-1) and *clec4n* (dectin-2) expression were determined by real-time PCR. (**c**) Alveolar macrophages were isolated by lavage from WT and KO mice 6 h after challenge with GFP-*C. albicans* and the % macrophages containing internalized GFP-*C. albicans* determined by microscopy. Results are mean ± SEM (*n* = 3–4). *p < 0.05 compared to cells from WT mice

Neutrophils and alveolar macrophages play an important role in host defense against *C. albicans*, however, despite the increase in neutrophil influx in cPLA_2_α^−/−^ mice the fungus was not completely cleared from these mice. Therefore the ability of these cells to recognize and kill *C. albicans* was determined. Alveolar macrophages and neutrophils were isolated from uninfected cPLA_2_α^+/+^ and cPLA_2_α^−/−^ mice, and their ability to bind and internalize GFP-*C. albicans* (recognition assay) and to kill the fungus was examined in vitro. The binding/internalization of GFP-*C. albicans* (unopsonized and opsonized) by cPLA_2_α^+/+^ and cPLA_2_α^−/−^ alveolar macrophages was similar but killing of GFP-*C. albicans* was ~25–30 % lower in cPLA_2_α^−/−^ macrophages (Fig. 7a). Alveolar macrophages from cPLA_2_α^+/+^ and cPLA_2_α^−/−^ mice expressed similar levels of the lectin receptors dectin-1 (*Clec7a*) and dectin-2 (*Clec4n*). Neutrophils from cPLA_2_α^−/−^ mice exhibited ~20–30 % less recognition and killing of GFP-*C. albicans* than cPLA_2_α^+/+^ neutrophils (Fig. 7b). Neutrophils from cPLA_2_α^+/+^ and cPLA_2_α^−/−^ mice expressed similar levels of dectin-1 but the levels of dectin-2 (*Clec4n*) were significantly higher in neutrophils from cPLA_2_α^−/−^ compared to cPLA_2_α^+/+^mice, although dectin-2 (*Clec4n*) expression was 10-fold lower than dectin-1 (*Clec7a*) in neutrophils. We also evaluated the number of GFP-*C. albicans* that were engulfed by alveolar macrophages in vivo, which were isolated by lavage from cPLA_2_α^+/+^ and cPLA_2_α^−/−^ mice 6 h after challenge with GFP-*C. albicans*. Alveolar macrophages from cPLA_2_α^−/−^ mice had significantly lower numbers of internalized GFP-*C. albicans* than macrophages from cPLA_2_α^+/+^ mice (Fig. 7b). The results suggest that the higher levels of *C. albicans* in cPLA_2_α^−/−^ mice may in part be due to a reduced capacity of alveolar macrophages and neutrophils to kill *C. albicans*.

## Discussion

cPLA_2_α is a highly conserved enzyme that is widely expressed throughout all tissues in mice and humans, and is rapidly activated by diverse agonists through common signaling pathways [1]. It is the only mammalian PLA_2_ that preferentially releases *sn-2* arachidonic acid from phospholipids and its role in initiating the production of eicosanoids is well documented [40, 41]. Identification of humans with cPLA_2_α deficiency has confirmed that it mediates eicosanoid production and functions in homeostatic processes important for human health [42–45]. cPLA_2_α has been implicated in regulating both normal physiological processes and disease pathogenesis in many organ systems from studies using cPLA_2_α^−/−^ mice, however, the specific mechanisms involved in many cases have not been elucidated [1, 46, 47]. In models of lung disease, cPLA_2_α^−/−^ mice are protected from pulmonary fibrosis, acute lung injury and allergic responses [48–50]. Since lung fibrosis and allergic lung responses are exacerbated in COX-1^−/−^ and COX-2^−/−^ mice but reduced in 5-LO^−/−^ mice, the results suggest that in certain pro-inflammatory disease states cPLA_2_α contributes to disease through a dominant role for pro-inflammatory leukotrienes [51–54]. By comparing cPLA_2_α^+/+^ and cPLA_2_α^−/−^ mice in this study, we are probing the primary mechanism for eicosanoid production in vivo in response to exposure of the lung to the opportunistic pathogen *C. albicans*. This model reflects the collective influence of lipid mediators resulting from cPLA_2_α activation in regulating innate immune responses. Immune competent mice are resistant to infection from intratracheal instillation of *C. albicans*, which is rapidly cleared from the lungs with minimal health effects due to contributions from both alveolar macrophages and neutrophils in host defense [35]. Our results suggest that cPLA_2_α contributes to innate immune defense mechanisms in the lung to control *C. albicans* infection and dampen inflammation.

cPLA_2_α^−/−^ mice do not clear *C. albicans* from the lung as efficiently as cPLA_2_α^+/+^ mice and exhibit greater signs of inflammation including excessive weight loss, increased production of pro-inflammatory cytokines and increased neutrophil recruitment to the lung. Pro-inflammatory cytokines (TNFα, IL1α, IL1β) are higher in cPLA_2_α^−/−^ than cPLA_2_α^+/+^ mice 6–24 h after *C. albicans* infection. In mouse models of bacterial pneumonia these cytokines are produced by alveolar macrophages from initial interaction with pathogens and signal to epithelial cells and neutrophils to mount responses to infection [55–57]. Alveolar macrophages, isolated 6 h after intratracheal instillation, contain engulfed GFP-*C. albicans* indicating that the fungi reach the alveoli shortly after instillation. Pro-inflammatory cytokines have been shown to induce the production of neutrophilic chemokines such CXCL1, which is higher in cPLA_2_α^−/−^ mice and correlates with the elevated neutrophil influx [56, 58]. *C. albicans* infection in cPLA_2_α^+/+^ mice leads to a small but significant increase in production of TNFα, IL1α and IL1β, and induces neutrophil influx, although at lower levels than in cPLA_2_α^−/−^ mice. It is likely that these innate immune responses in cPLA_2_α^+/+^ mice are important for host defense resulting in clearance of *C. albicans* from the lung. It has been shown that TNFα, IL1α and IL1β are important for host defense against invasive *C. albicans* infection in mice [59, 60]. However, the exaggerated responses to *C. albicans* infection in cPLA_2_α^−/−^ mice point to an important role for cPLA_2_α in regulating the balance of cytokines produced for effective microbial clearance without excess inflammation that may cause tissue injury and dissemination of *C. albicans* from the lung. This may in part be due to higher levels of PGE_2_ in cPLA_2_α^+/+^ mice since prostaglandins suppress the production of TNFα, IL1α and IL1β [15, 61–63]. PGE_2_ is also important in maintaining endothelial barrier function, promoting wound healing and inhibiting neutrophil migration [64]. PGI_2_ also has anti-inflammatory properties [3]. Our results show relatively high levels of endogenous PGI_2_ in BALF suggesting constitutive production perhaps by vascular endothelial cells and smooth muscle cells reflecting its important role in maintenance of the vasculature [65]. PGI_2_ levels were similar in BALF from cPLA_2_α^+/+^ and cPLA_2_α^−/−^ mice, and not increased by *C. albicans* infection, suggesting another PLA_2_ is involved in its production and that it is not involved in the phenotypic differences observed during *C. albicans* infection.

Of the cytokines measured in BALF, IL6 showed the greatest increase in cPLA_2_α^−/−^ mice early after *C. albicans* instillation reaching levels 10-fold higher than in cPLA_2_α^+/+^ mice. IL6 is an indicator of disease severity, reflecting the more pronounced effect of *C. albican*s on the health of cPLA_2_α^−/−^ compared to cPLA_2_α^+/+^ mice, which show only a small increase in IL6 production [66]. IL6 is considered a pleiotropic cytokine made by immune and stromal cells in response to diverse agonists that has a homeostatic function and regulates immunity [67]. IL6 regulates the recruitment of leukocytes during infection and may contribute to the higher neutrophil influx in cPLA_2_α^−/−^ mice [67, 68]. Although IL6 can be induced by prostaglandins, its higher level in cPLA_2_α^−/−^ mice suggests that it is directly made by cells in response to *C. albicans* perhaps through the early production of TNFα, IL1α, and IL1β [66, 69, 70]. In contrast to the results of this study, cPLA_2_α^−/−^ mice are protected during *Pseudomonas aeruginosa* lung infection that correlates with decreased IL6 production [71]. Therefore, cPLA_2_α can exacerbate infection or have a protective role in the lung depending on the type of pathogen.

Leukotrienes also regulate immunity in the lung during infection by promoting trafficking of neutrophils, T lymphocytes, dendritic cells and vascular permeability [2, 7]. Mice deficient in leukotriene production are more susceptible to bacterial (*Klebsiella pneumonia, Mycobacterium tuberculosis*) and fungal (Histoplasmosis) lung infection showing impaired microbial clearance and survival [72–74]. However there are differences in the responses of leukotriene-deficient mice to bacterial and fungal infection. Following bacterial challenge, 5-LO^−/−^ mice have reduced neutrophil influx in the lung [72]. However, *Histoplama capsulatum* lung infection in 5-LO^−/−^ mice results in increased neutrophil recruitment and greater production of pro-inflammatory cytokines than in wild type mice, as we observed in *C. albicans*-infected cPLA_2_α^−/−^ mice. Leukotrienes regulate innate immune responses in part by enhancing alveolar macrophage phagocytosis and microbial killing [72, 74].

Our results demonstrate that alveolar macrophages and neutrophils from uninfected cPLA_2_α^−/−^ mice have a reduced capacity to kill *C. albicans* than cells from cPLA_2_α^+/+^ mice. We previously reported that *C. albicans* poorly activates cPLA_2_α in alveolar macrophages from cPLA_2_α^+/+^ mice and induces very little eicosanoid production, although it is enhanced by priming with GM-CSF due to increased expression of dectin-1 [12]. Therefore it is not likely that this inherent difference in the killing capacity of alveolar macrophages from uninfected cPLA_2_α^+/+^ and cPLA_2_α^−/−^ mice is due to production of endogenous eicosanoids during the killing assay in vitro. The basis for this inherent difference in *C. albicans* killing is not known but the lack of eicosanoids during development of cPLA_2_α^−/−^ mice may affect gene expression that influences killing of *C. albicans*. The results also showed that alveolar macrophages isolated from cPLA_2_α^−/−^ mice 6 h after instillation of GFP-*C. albicans* have fewer engulfed GFP-*C. albicans* than macrophages from cPLA_2_α^+/+^ mice. It is likely that cells are primed by cytokines in vivo to enhance production of eicosanoids and regulate killing of *C. albicans*.

A role for the epithelium during *C. albicans* lung infection is suggested by results showing that cPLA_2_α^−/−^ mice have higher levels of CCL20 and CSF2 than cPLA_2_α^+/+^ mice. During lung infection CCL20 and CSF2 (GM-CSF) are derived from lung epithelium and contribute to recruitment of dendritic cells and neutrophils [55, 58, 75]. The lung epithelium may also contribute to production of pro-inflammatory cytokines since *C. albicans* stimulates oral and vaginal epithelial cells to produce chemokines and cytokines including IL1α, IL1β and TNFα [76, 77]. Although this has not been investigated in lung epithelial cells, there may be a local immune response at the lung mucosa for combating *C. albicans* in cPLA_2_α^+/+^ mice. It is interesting that *C. albicans* disseminates to the kidney in cPLA_2_α^−/−^ mice suggesting there is damage to the epithelial/endothelial barrier possibly due to the increased inflammation. Since alveolar epithelium damage can be sensed by alveolar macrophages this may lead to heightened pro-inflammatory responses as we observed in cPLA_2_α^−/−^ mice [78]. The results suggest that cPLA_2_α activation is an important mechanism for regulating the function of immune and stromal cells in the lung to protect from *C. albicans* infection.

## Conclusions

This study demonstrates that cPLA_2_α plays a role in protecting the lung from *C. albicans* infection. Since production of lipid mediators occurs rapidly in response microbial infection we focused on how this pathway regulates the early innate immune responses to *C. albicans* in the lung in an attempt to assess the more immediate effects of this pathway. The results suggest that cPLA_2_α contributes to lung homeostasis and the immunosuppressive environment in the lung. There may be tonic pattern receptor signaling resulting in cPLA_2_α activation and lipid mediator production in the lung by low-level colonization or exposure to commensal organisms such as *C. albicans* from the oral cavity. This promotes clearance of the relatively avirulent commensal fungus that limits infection and inflammation preventing more pathogenic effects. It is likely that the balance of products from both cyclooxygenase and lipoxygenase pathways is important in immune surveillance in the lung contributing to mucosal integrity and the function of phagocytes for efficient clearance of infectious agents and regulating the extent of inflammation.

## Abbreviations

5-LO, 5-lipoxygenase; BALF, bronchoalveolar lavage fluid; CFU, colony forming units; COX, cyclooxygenase; cPLA_2_α, Group IVA cytosolic phospholipase A_2_; DAPI, 4’,6-diamidino-2-phenylindole; FC, flow cytometry; GFP, green fluorescent protein; GM-CSF, granulocyte macrophage colony-stimulating factor; HPLC, high performance liquid chromatography; XTT, 2,3-bis(2-methoxy-4-nitro-5-sulfophenyl)-5-[(phenylamino)carbonyl]-2H-tetrazolium hydroxide

## Additional files

Additional file 1: Figure S1. Flow cytometry gating strategy for cell identification in lung digests from cPLA_2_α^+/+^ (WT) and cPLA_2_α^−/−^ (KO) mice challenged with *C. albicans* for 24 h. Cells were isolated from enzymatically digested mouse lungs, and after exclusion of doublets and debris, immune cells were identified by CD45 staining. A sequential gating strategy was used to identify populations expressing specific markers: **a** alveolar macrophages (AM) (CD45^+^ CD24^−^ CD11b^−^ SiglecF^+^), (**b**) tissue macrophages (TM) (CD45^+^ CD24^−^ CD11b^+^), (**c**) neutrophils (PMN) (CD45^+^ CD11b^+^ Ly6G^+^) and (**d**) CD11b^+^ dendritic cells (CD11b^+^ DCs) (CD45^+^ MHCII^+^ CD11c^+^ CD11b^+^). (TIF 954 kb) Additional file 2: Figure S2. Expression of cytokines and chemokines in lung tissue from cPLA_2_α^+/+^ and cPLA_2_α^−/−^ mice during *C. albicans* infection. Real-time PCR was carried out using the Mouse Cytokines & Chemokines RT^2^ Profiler PCR Array to compare expression in lungs of cPLA_2_α^−/−^ (KO) and cPLA_2_α^+/+^ (WT) mice challenged with saline or 10^6^ *C. albicans* (CA) for 12 and 24 h (*n* = 6-10 mice/group in 3–5 experiments). **P* < 0.05 compared to WT saline control; ^ϕ^ *P* < 0.05 compared to WT saline control, ^#^ *P* < 0.05 compared to KO saline control; ***P* < 0.05 compared to WT with CA. (TIF 859 kb)
