# Supplementary figures and images for: Cytosolic phospholipase A2 contributes to innate immune defense against Candida albicans lung infection
Source: BMC Immunol. 2016 Aug 8;17:27. doi: 10.1186/s12865-016-0165-9 (PMC4977843; doi:10.1186/s12865-016-0165-9)

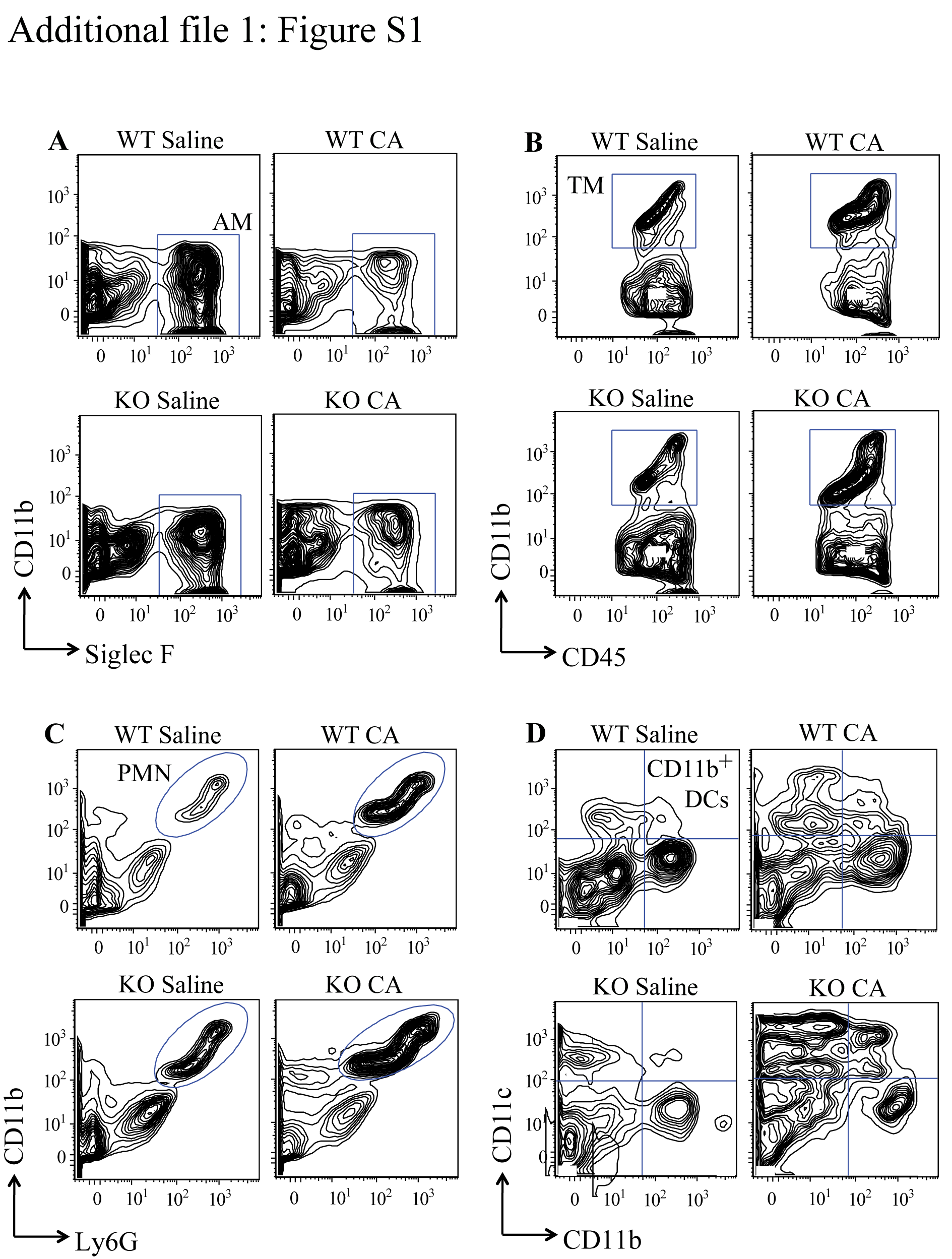

Supplement: Additional file 1: Figure S1. — Flow cytometry gating strategy for cell identification in lung digests from cPLA2α+/+ (WT) and cPLA2α−/− (KO) mice challenged with C. albicans for 24 h. Cells were isolated from enzymatically digested mouse lungs, and after exclusion of doublets and debris, immune cells were identified by CD45 staining. A sequential gating strategy was used to identify populations expressing specific markers: a alveolar macrophages (AM) (CD45+ CD24− CD11b− SiglecF+), (b) tissue macrophages (TM) (CD45+ CD24− CD11b+), (c) neutrophils (PMN) (CD45+ CD11b+ Ly6G+) and (d) CD11b+ dendritic cells (CD11b+ DCs) (CD45+ MHCII+ CD11c+ CD11b+). (TIF 954 kb) [file 12865_2016_165_MOESM1_ESM.tif]

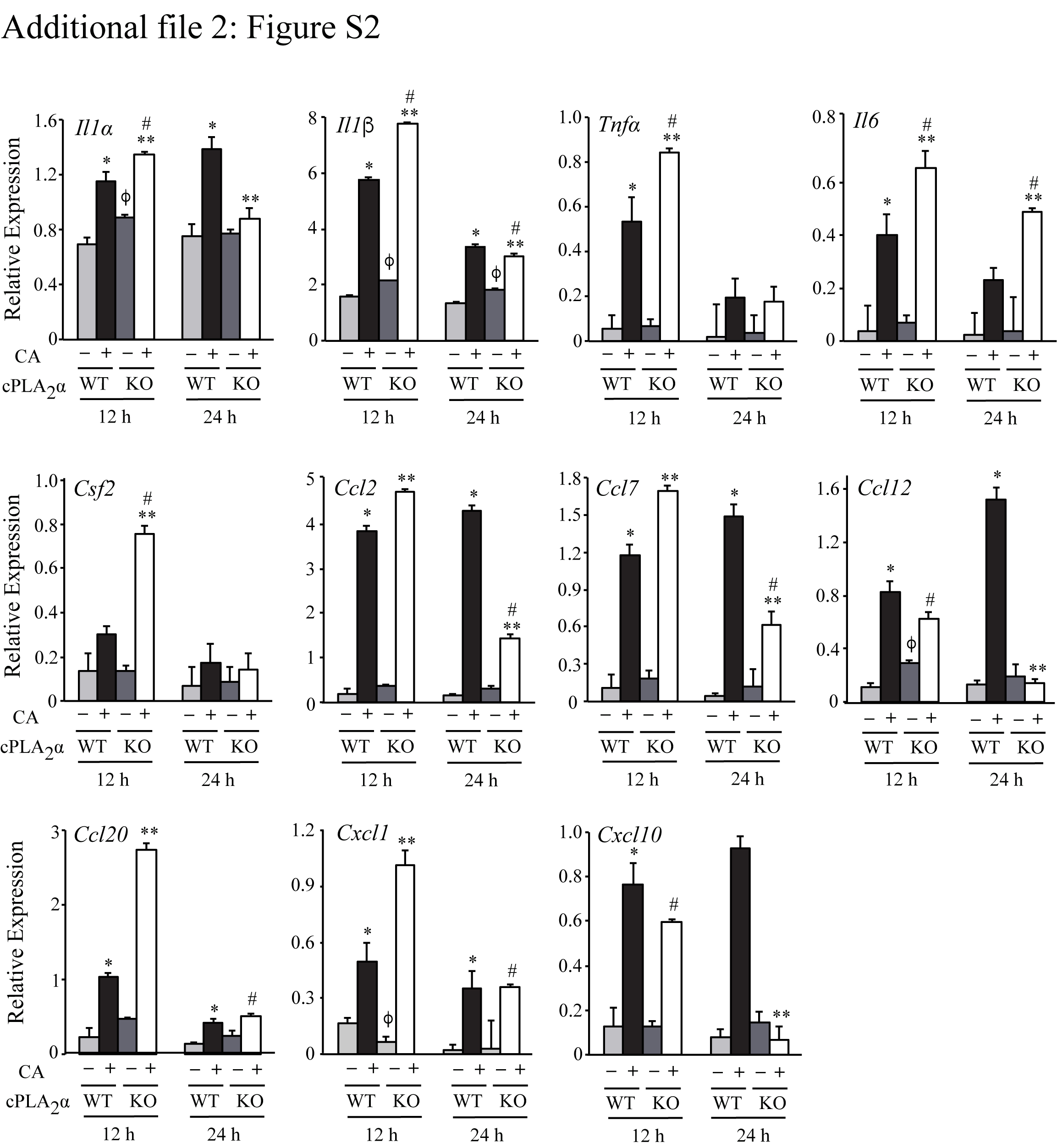

Supplement: Additional file 2: Figure S2. — Expression of cytokines and chemokines in lung tissue from cPLA2α+/+ and cPLA2α−/− mice during C. albicans infection. Real-time PCR was carried out using the Mouse Cytokines & Chemokines RT2 Profiler PCR Array to compare expression in lungs of cPLA2α−/− (KO) and cPLA2α+/+ (WT) mice challenged with saline or 106 C. albicans (CA) for 12 and 24 h (n = 6-10 mice/group in 3–5 experiments). *P < 0.05 compared to WT saline control; ϕ P < 0.05 compared to WT saline control, # P < 0.05 compared to KO saline control; **P < 0.05 compared to WT with CA. (TIF 859 kb) [file 12865_2016_165_MOESM2_ESM.tif]
